# Management of a fractured and retained plastic stent during endoscopic ultrasound-guided hepaticogastrostomy

**DOI:** 10.1055/a-2512-3670

**Published:** 2025-01-21

**Authors:** Yasuhiro Kuraishi, Akira Nakamura, Takeji Umemura

**Affiliations:** 1 Department of Gastroenterology, Shinshu University Hospital, Matsumoto, Japan

Plastic stents used for endoscopic ultrasound-guided hepaticogastrostomy (EUS-HGS) are typically connected to an inner catheter, which enables retraction if repositioning is necessary. We present a case where the stent–inner catheter connection detached during EUS-HGS, resulting in stent fracture and intrahepatic retention during retraction, and describe the successful management of this complication.

A 74-year-old man with obstructive jaundice from pancreatic cancer underwent EUS-HGS, with a fully covered self-expandable metallic stent (FCSEMS) placed in the B3 duct ( Fig. 1 ). The stent dislodged after 2 months, causing acute cholangitis. Following temporary percutaneous biliary drainage at another hospital, he was referred to our institution for repeat EUS-HGS.

**Fig. 1:**
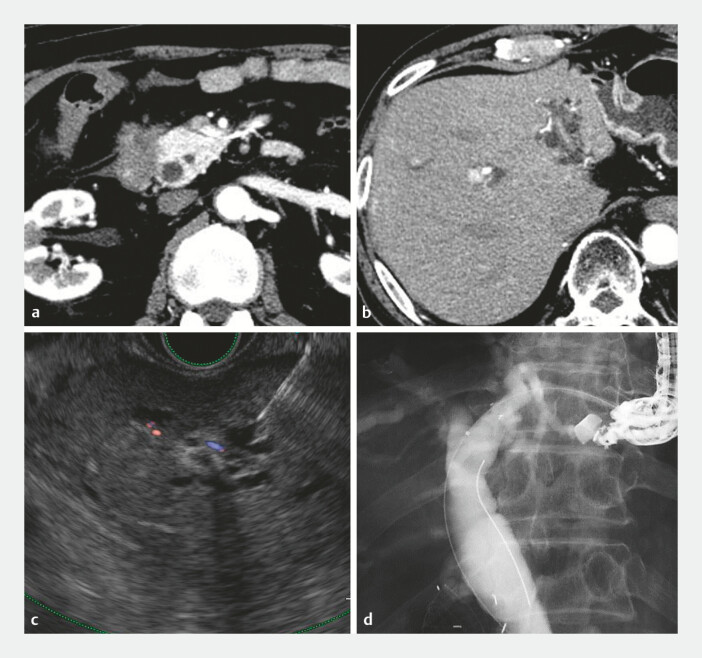
Imaging studies. **a** Computed tomography (CT) showed pancreatic head cancer, which led to distal bile duct stricture and duodenal stenosis. **b** CT revealed intrahepatic duct dilation and left hepatic lobe hypoplasia. **c** Endoscopic ultrasound-guided hepaticogastrostomy was performed for biliary drainage using an oblique-viewing echoendoscope. The B3 duct was punctured with a 19-G needle. **d** A 6-mm fully covered self-expandable metallic stent (HANAROSTENT Biliary Full Cover Benefit; Boston Scientific, Marlborough, Massachusetts, USA) was placed in the B3 duct.

Puncture sites were limited due to hypoplasia of the left hepatic lobe and scarring from the prior intervention. We punctured the proximal left hepatic duct using a 19-G needle, but encountered difficulty with the thickened bile duct wall ( Fig. 2 , Video 1 ). After inserting a 0.025-inch guidewire and dilating the fistula with a drill dilator, we employed a double-guidewire technique 1 2 , adding a second guidewire to assist with possible challenges with stent placement. Indeed, the plastic stent failed to traverse the thickened bile duct wall, and the stent–inner catheter connection detached during stent retraction. Leaving one guidewire in place, we attempted stent retrieval using forceps, but the distal tip fractured and was retained within the fistula. We opted to leave the fractured stent in place, expand the fistula with a 4-mm balloon dilator, and place a 6-mm FCSEMS.

**Fig. 2:**
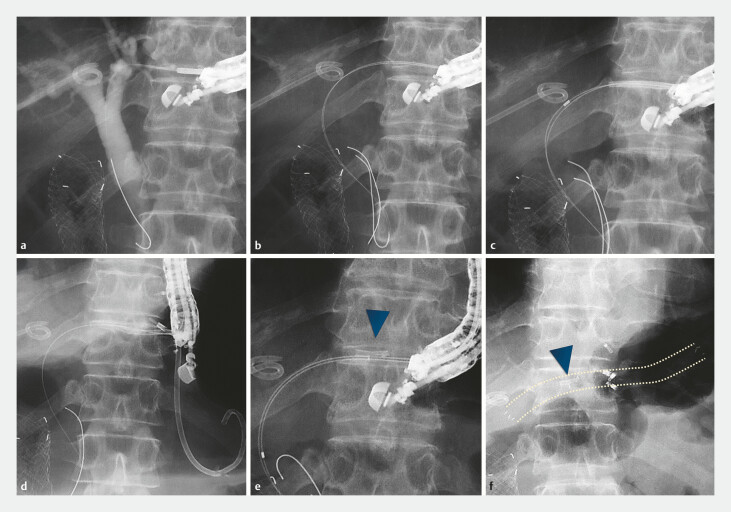
**a** In the second endoscopic ultrasound-guided hepaticogastrostomy procedure, the proximal left hepatic duct was punctured with a 19-G needle, and a 0.025-inch guidewire was inserted into the bile duct. **b** Owing to the anticipated difficulty in stent placement from inflammation-induced bile duct wall thickening, a double-guidewire technique was employed by inserting an additional guidewire through a double-lumen catheter. **c** Attempts to place a plastic stent (ThroughPass TYPE IT; Gadelius Medical, Tokyo, Japan) failed because the thickened bile duct wall could not be traversed. Upon stent retraction for further fistula dilation, the stent-inner catheter connection detached. **d, e** Leaving one guidewire in the bile duct, we attempted to retrieve the stent using a forceps, but the distal stent tip fractured and remained lodged within the fistula. Arrowhead indicates the fractured stent. **f** As removal of the fractured stent was deemed impossible at this time, we placed a 6-mm fully covered self-expandable metallic stent (HANAROSTENT Biliary Full Cover Benefit) alongside the fractured stent (arrowhead). Dotted lines show the path of the metallic stent.

Detachment of the stent–inner catheter connection posed a significant challenge during endoscopic ultrasound-guided hepaticogastrostomy. However, the use of a double-guidewire technique, which provided a safety guidewire, enabled successful biliary drainage. Despite the complication of a fractured stent retained within the fistula, creating a sufficiently wide fistula with a metallic stent allowed for successful retrieval of the fractured stent in a subsequent procedure.Video 1

After FCSEMS removal 2 weeks later, a sufficiently wide fistula had formed, enabling retrieval of the fractured stent endoscopically with biopsy forceps and placement of a new plastic stent ( Fig. 3 ).

**Fig. 3:**
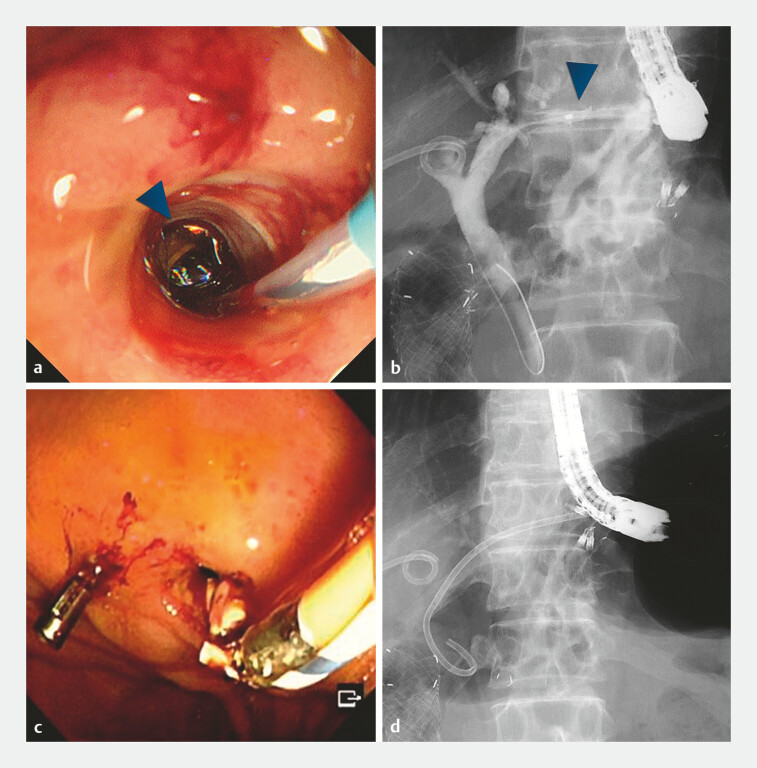
Retrieval of the fractured stent. **a** A sufficiently wide fistula was observed after removing the metallic stent, allowing for endoscopic visualization of the fractured stent (arrowhead) within the fistula. **b** Cholangiography confirmed the fully formed fistula with the retained fractured stent (arrowhead) inside. No contrast medium leakage outside the fistula was observed. **c** The fractured stent was successfully retrieved using biopsy forceps. **d** A new plastic stent was placed through the fistula.

Despite the complication of stent–inner catheter connection detachment, a double-guidewire technique, which provided a safety guidewire, facilitated biliary drainage. The retained fractured plastic stent was successfully retrieved after creating a wide fistula with an FCSEMS.

Endoscopy_UCTN_Code_CPL_1AL_2AD

## Notes

Endoscopy E-Videos https://eref.thieme.de/e-videos
*E-Videos* is an open access online section of the journal *Endoscopy* , reporting on interesting cases and new techniques in gastroenterological endoscopy. All papers include a high-quality video and are published with a Creative Commons CC-BY license. Endoscopy E-Videos qualify for HINARI discounts and waivers and eligibility is automatically checked during the submission process. We grant 100% waivers to articles whose corresponding authors are based in Group A countries and 50% waivers to those who are based in Group B countries as classified by Research4Life (see: https://www.research4life.org/access/eligibility/ ). This section has its own submission website at https://mc.manuscriptcentral.com/e-videos .
